# Synthesis and Photocatalytic Performance of ZnO/Bone Char Composite

**DOI:** 10.3390/ma11101981

**Published:** 2018-10-15

**Authors:** Puqi Jia, Hongwei Tan, Kuiren Liu, Wei Gao

**Affiliations:** 1 College of Earth and Environmental Sciences, Lanzhou University, Lanzhou 730000, China; jpq@lzu.edu.cn; 2 Department of Nonferrous Metallurgy, School of Metallurgy, Northeastern University, Shenyang 110819, China; liukr@smm.neu.edu.cn; 3 Department of Chemical and Materials Engineering, The University of Auckland, Auckland 1142, New Zealand; htan393@aucklanduni.ac.nz

**Keywords:** ZnO, bone char, uniform layer, UV–visible absorbance, photocatalysis

## Abstract

ZnO/bone char (ZnO/BC) composites were successfully synthesized by the precipitation of a ZnO precursor on pyrolytic bone char. The effects of bone char size, mass ratio of ZnO to BC, and molar ratio of ZnO to triethylamine (TEA) on the microstructure, specific surface area, and light absorbance of ZnO/BC were studied. The photocatalytic property of ZnO/BC was evaluated by the degradation of methylene blue. Results show that with a uniform nano-ZnO particle layer distributed evenly on the bone char surface, ZnO/BC has the strongest light absorbance and can effectively degrade methylene blue. The photocatalytic performance of ZnO/BC is related to the light absorbance of the photocatalyst, as well as the amount and distribution state of the loaded ZnO. This study indicates that bovine bone waste can be used as a nano-photocatalyst carrier to prepare photocatalytic composites, which is not only a good way to clean wastewater but also an ideal solution to utilize animal bone waste.

## 1. Introduction

Billions of animals are used daily to meet the dietary needs of humans and carnivores. Bone is one of the appendant byproducts. If not handled in time, it becomes solid waste, breeds bacteria, endangers human health, and pollutes the environment. Bone products are worth $4000 to the total output value after processing a ton of raw bone materials. The largest daily-output bone species in the world are beef bones, lamb bones, and pork bones. Slaughter plants usually process bones into bone grains, then sell them to agricultural and sideline product manufacturing companies for further processing. The products extracted from bones reach 7 major categories, including 300 varieties, amongst which the most common products are bone soup, bone oil, bone marrow extract, bone meal, and bone flavor. After disinfection by boiling, the bone particles can also be used as fertilizer for green plants.

Bone char can be obtained after removing organics by pyrolysis; it is mainly composed of hydroxyapatite and commonly used as the raw material for the high-grade bone china of ceramics. With the advantages of thermostability, alkali resistance, water insolubility, large specific surface area, good adsorption ability, nontoxicity, and recyclability, bone char has been widely used as an adsorbent for refining sugar [1] and fish oil [2] and removing heavy metal ions [3,4] and fluoride [5]. Recently, some researchers also applied it to adsorb 17-estradiol [6], endotoxin [7], and methylene blue [8,9].

Green degradation technology uses photocatalytic oxidation to oxidize toxic organic compounds and reduce heavy metal ions [10,11,12]. It plays a key role in solving serious environmental problems, such as contaminated river, air, and soil. Nanosized photocatalyst powder is used as a photocatalyst; however, it tends to agglomerate, reducing the photocatalytic efficiency. Another shortcoming is that it is difficult to recover, resulting in secondary environmental pollution. Large-sized supported photocatalysts can be synthesized by combining large particle sizes of millimeter-scale bone char with nano photocatalytic materials. This technique not only improves the high value-added bone waste, but also solves the environmental pollution problem.

It was reported that the combination of photocatalysis with adsorption by mixing nano ZnO photocatalyst and bone char (BC) particles increased the removal efficiency of a formaldehyde pollutant [13]. However, nano ZnO particles were not distributed evenly on the surface of the bone char through physical mixing. A uniform nano ZnO particle layer loaded on the surface of bone char is expected to yield perfect photocatalytic performance. In this paper, to optimize the micromorphology and photocatalytic performance of ZnO/BC, ZnO/BC was prepared by depositing ZnO precursor on bone char surface. The size of bone char, mass ratio of ZnO to BC, and molar ratio of ZnO to triethylamine (TEA) were investigated. Their effects on the microstructures, specific surface areas, and absorbance spectra of ZnO/BC photocatalyst were studied. The degradation of methylene blue from water was also carried out.

## 2. Materials and Methods

Bovine bone was sawed into 2–3 cm bone slices by handsaw and washed in boiled water until fat and residual protein pieces were removed completely, then, the bone slices were crushed into bone particles with a size of 0.25–0.50 mm or 0.50–0.80 mm, according to our previous study [14]. The bone particles were pyrolyzed at 400 °C for 2 h in a furnace. ZnO/BC composites were prepared as we reported before [15], namely, zinc acetate dehydrate was added to 55 °C absolute ethanol solution and dissolved with a little of the capping agent triethylamine (TEA). Zinc acetate dehydrate and TEA were mixed at a molar ratio of 1:1 or 2:1 to form 0.2 mol/L of nano ZnO precursor sol (zinc hydroxide) in absolute ethanol solution. The pH value of ZnO precursor sol was 8.4. Bone char was dipped in the ZnO precursor solution to produce 1:5.5, 1:11, or 1:16.5 of *m*(ZnO):*m*(BC) for 3 h. Then, zinc hydroxide and bone char combined with each other through hydrogen bonding. The as-prepared samples were dried at room temperature through the volatilization of absolute ethanol and acetic acid, and further at 110 °C in an oven for 8 h, followed by sintering at 400 °C for 1 h to obtain ZnO/BC samples. Table 1 shows the sample labels.

The crystals structure of ZnO/BC samples were identified by using an X-ray diffractometer (Bruker, D2 Phaser, Billerica, MA, USA). The microstructures were characterized by an SEM (JEOL, JSM-7800F, Tokyo, Japan). The specific surface areas of bone char and ZnO/BC samples were analyzed by a surface area instrument (Micromeritics, 3 FLEX, Norcross, GA, USA). The ultraviolet–visible absorbance spectra of the ZnO/BC photocatalyst were measured on a UV–vis spectrophotometer (Shimadzu, UV-2550, Kyoto, Japan) using BaSO_4_ as the reflectance standard.

The photocatalytic performance of ZnO/BC (4 g/L) was evaluated through the photocatalytic degradation of methylene blue (MB, 4.48 mg/L) under simulated solar irradiation (OSRAM, ULTRA-VITALUX, 300 W, Munich, Germany), and the reaction system was cooled by a circulating water bath to maintain room temperature. Oxygen was introduced into the reaction system at a rate of 0.4 L/min. Dark adsorption was carried out without a light illuminator. The concentrations of the MB solution were measured using a UV–vis spectrometer (Perkin Elmer, Lambda 35, Waltham, MA, USA) at 664 nm during the reaction.

## 3. Results and Discussion

**Figure 1:**
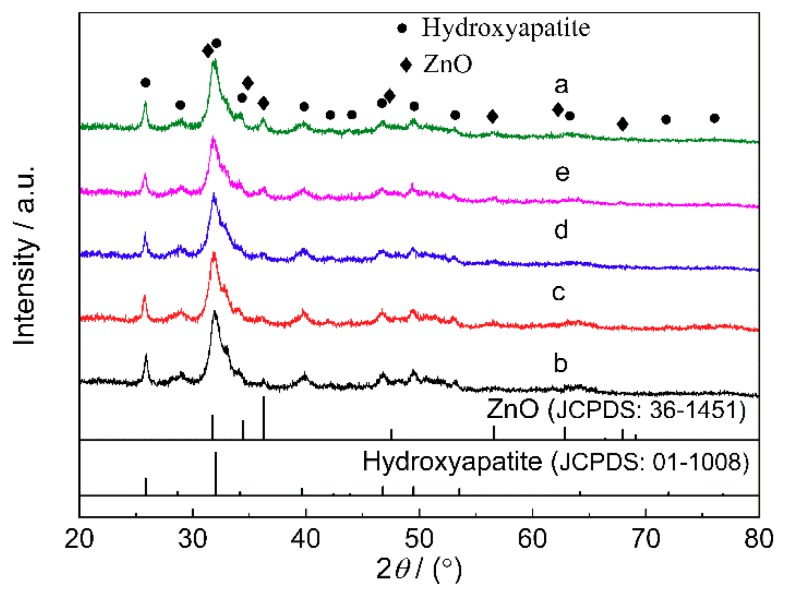
XRD patterns of ZnO/BC samples.

The XRD patterns of the obtained ZnO/BC samples are shown in Figure 1. All detectable peaks can be indexed to the hydroxyapatite and ZnO hexagonal wurtzite structure. Hexagonal wurtzite ZnO is an effective structure for photocatalysis. Because the mass ratios of ZnO to bone char were less than 1:5.5, the peak intensities of ZnO are much weaker than those of bone char. The peak intensity of ZnO at 36.25° (2θ) in sample a is the highest of all samples because this sample had the highest mass ratios of ZnO to bone char.

**Figure 2:**
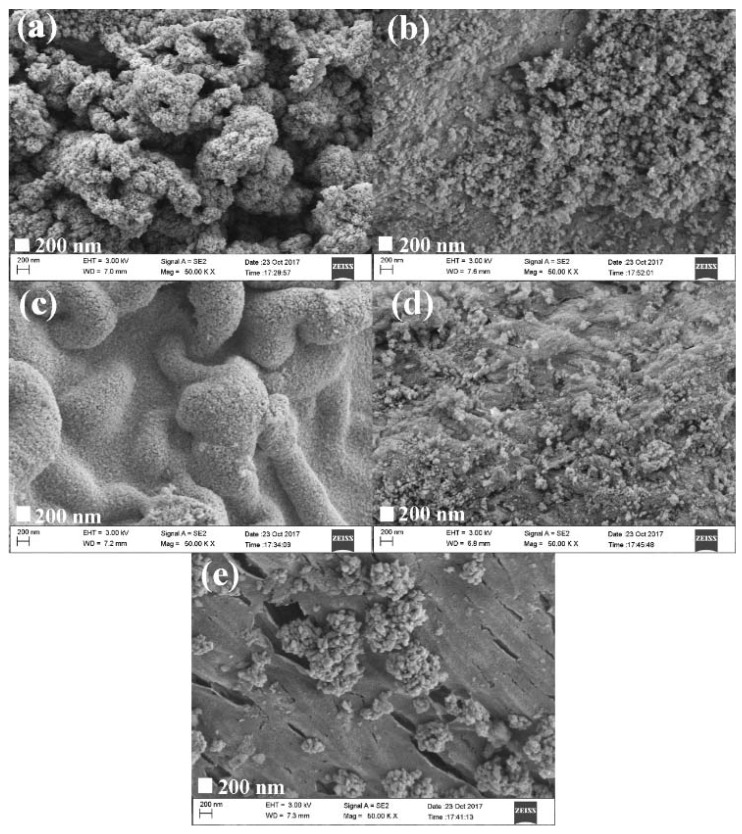
SEM images of ZnO/BC samples: (**a**) sample a, (**b**) sample b, (**c**) sample c, (**d**) sample d, and (**e**) sample e.

Figure 2 shows the SEM images of ZnO/BC. Sample a (Figure 2a) and sample b (Figure 2b) show a large number of stacked ZnO particles loaded on the bone char surface due to the large mass ratio of ZnO to bone char. The XRD peak intensity of ZnO at 36.25° (2θ) in sample b was weaker than that of sample a because some bare bone char decreased a proportion of ZnO exposed to X-ray. Reducing the amount of ZnO precursor by half, ZnO particles with a size of about 30 nm were uniformly distributed on the bone char surface by layer in sample c (Figure 2c). Further reducing the amount of ZnO precursors, the ZnO particles in sample d (Figure 2d) were distributed sparsely on the bone char surface. The ZnO precursor could not be dispersed uniformly in absolute ethanol solution by reducing the amount of capping agent, triethylamine; eventually, the ZnO in sample e (Figure 2e) easily agglomerated into large particles.

Table 2 lists the specific surface areas and average pore sizes of the ZnO/BC samples. The average pore sizes of the ZnO/BC samples were about 12–14 nm, namely mesopores. The specific surface areas of ZnO/BC listed in Table 2 were smaller than those of the bone char samples, because the loaded nano ZnO particles could cover some of the bone char surface area. Of all ZnO/BC samples, sample e had the largest exposed bone char surface, so it had the largest specific surface area. The partly exposed bone char surface and the agglomerated multilayered ZnO particles of sample b gave it a slightly larger specific surface area than sample a.

As shown in Figure 2, a large amount of ZnO particles were supported on the bone char surface of sample a owing to the large mass ratio of ZnO to BC and the strong hydrogen bonds generated in the precursor solution. However, the nano ZnO particles agglomerated after sintering, forming obvious holes due to the large surface energy. The specific surface areas of the bone char samples for the sizes of 0.25–0.50 mm and 0.50–0.80 mm were 119.2 m^2^/g and 116.0 m^2^/g, respectively, containing mainly mesopores (2–50 nm) [6]. The specific surface area of the bone char in sample b was smaller than that in sample a, implying that the slightly lower bone char surface energy decreased the amount of adsorbed ZnO and weakened the agglomeration of ZnO. Meanwhile, the specific surface area of sample b was bigger than that of sample a because of some bare mesopores and micropores on the bone char surface. The exposed bone char surface area of sample d was more than that of sample b and sample a, but the smallest amount of ZnO supported on the surface brought about a relatively even distribution, resulting in the smallest contribution of the nano ZnO particles to the specific surface area of the ZnO/BC composite, so sample d had the smallest specific surface area among the three samples. The bone char surface in sample c was smoothly and compactly covered by a uniform nano ZnO particle layer, causing it to have the smallest specific surface area. In summary, the specific surface areas of the ZnO/BC samples followed the order of: sample e > sample b > sample a > sample d > sample c.

**Figure 3:**
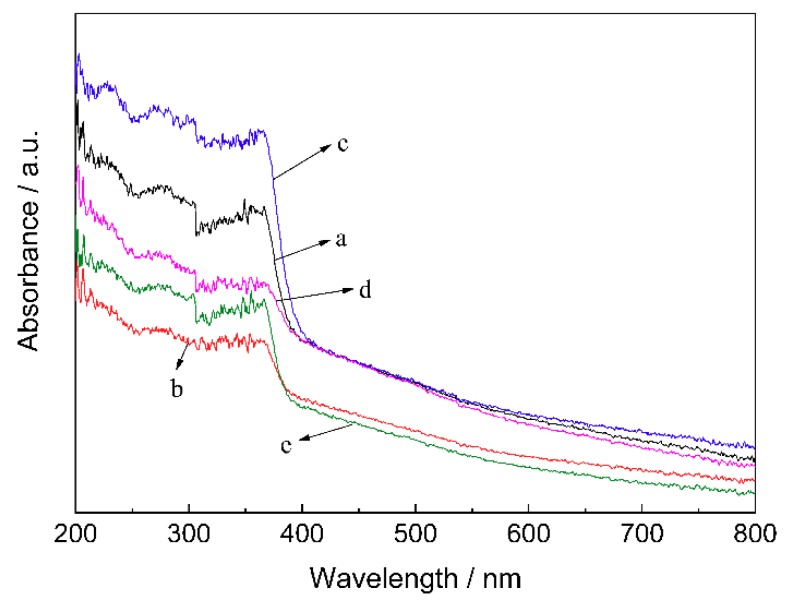
UV–vis absorbance spectra of different samples.

Figure 3 shows the UV–vis absorbance spectra of the ZnO/BC samples. The weak light absorbance of sample b and sample e was due to the fact that the agglomerated ZnO particles on the bone char surface could severely hinder the photon energy absorption of ZnO loaded in the bottom. The uniformly distributed nano-ZnO particles on the bone char surface increased the amount of ZnO exposed to light, enhancing the light absorbance of sample c. The ZnO under the hole and shadow of sample a made the amount of ZnO exposed to light less than that of sample c, weakening the light absorbance. Also, the low-proportion coverage of ZnO in sample d caused lower light absorbance.

Because ZnO is not stable when the pH level is less than 5 or more than 11 [16], the initial pH level of the MB solution was set at 10.4. In this situation, the surface hydroxy of bone char occured deprotonation and then formed negatively charged surface on the bone char (mainly hydroxyapatite) could quickly adsorb positive MB ions through strong electrostatic attraction [14]. For the photocatalysis process, the photogenerated holes produced by light irradiation migrate to the ZnO surface and act with the adsorbed MB nearby. Studies have shown that the added oxygen, acting as an electron acceptor, produces a higher reaction rate and photocatalytic capacity than with only the photocatalyst or oxygen [17,18]. Therefore, the photocatalytic experiments in this work were conducted under oxygen-rich conditions. The self-degradation rate of MB under simulated solar irradiation for 120 min was about 15.5%.

**Figure 4:**
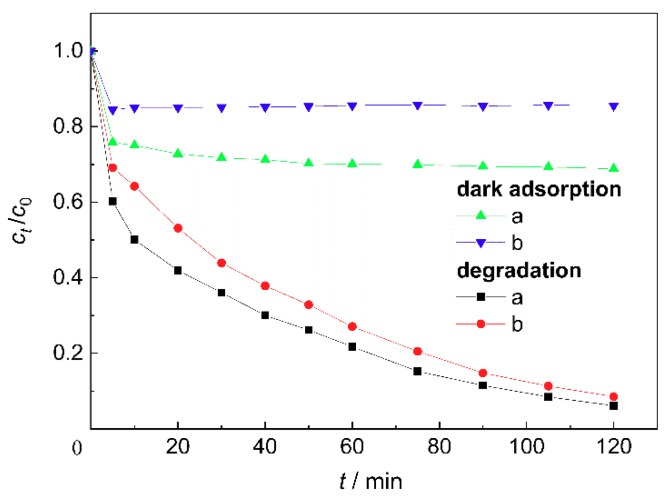
Effect of bone char size of ZnO/bone char on the dark adsorption and degradation of methylene blue.

Figure 4 shows the dark adsorption and degradation efficiencies of sample a and sample b for MB. The adsorption efficiency of sample a for MB was larger than that of sample b due to the contribution of adsorption sites by aggregated nano-ZnO particles in a loose and hollow state, as shown in Figure 2a. The ZnO particles below the hole shadow could not participate in photocatalytic reactions, as they were unexposed to light, resulting in the lower photocatalytic efficiency of sample a.

**Figure 5:**
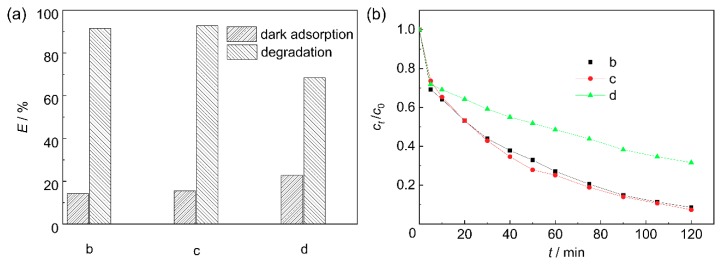
Effect of mass ratio of ZnO to bone char of ZnO/bone char photocatalyst on: (**a**) dark adsorption and degradation, and (**b**) degradation behavior for methylene blue.

Figure 5 shows the effect of mass ratios of ZnO to bone char on the dark adsorption and degradation efficiencies of ZnO/BC for MB. The contribution to photocatalysis was related to not only the light absorbance of the photocatalyst, but also the amount and distribution state of ZnO. Sample d had the highest adsorption efficiency and the lowest photocatalytic efficiency for MB, because sample d had the largest number of active bone char adsorption sites and the smallest proportion of loaded ZnO of the three samples shown (b, c, and d). The photocatalytic efficiency of sample b was close to that of sample c, because the extra ZnO loaded at the hole bottom could not receive light irradiation (see Figure 2b). The uniform nano-ZnO particle layer in sample c distributed evenly and made full use of light irradiation, contributing well to photocatalysis.

**Figure 6:**
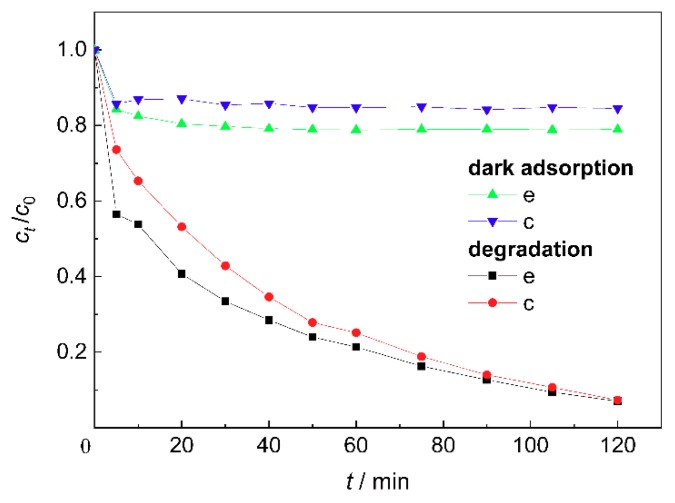
Effect of molar ratio of ZnO to TEA on the photocatalytic performance of ZnO/bone char.

Figure 6 shows the dark adsorption and degradation efficiencies of MB by the ZnO/BC samples based on different molar ratios of ZnO to TEA. The adsorption efficiency of sample e for methylene blue was higher than that of sample c due to the barer bone char surface, i.e., more adsorption sites, as shown in Figure 2e. After the deduction of adsorption, the lower photocatalytic efficiency of sample e was related to its weak light absorbance, and the growth and agglomeration of nano-ZnO particles, which inhibited the effective separation of photogenerated electrons and holes to some extent.

## 4. Conclusions

Bone char containing mainly hydroxyapatite (HAP) was produced by pyrolyzing bovine bone waste at 400 °C. ZnO/BC samples were synthesized using a process to adsorb ZnO precursor onto the surface of bone char. The effects of bone char size and the content of ZnO loading on the photocatalytic performance were studied. The light absorbance and the distribution of ZnO on the bone char were investigated to explain their effects on the photocatalytic efficiency. The experimental results indicate that the sample with a relatively large bone char size and suitable ZnO loading (sample c) possesses a uniform nano-ZnO particle layer and the strongest UV–visible absorbance and can effectively degrade methylene blue. Overall, this project indicates that bone char can be used as a good nano-photocatalyst supporter for the efficient degradation of environmental pollutants.

## Figures and Tables

**Table 1:** ZnO/bone char (BC) samples obtained by different synthesis parameters.

Sample ^1^	Particle Size of Bone Char/mm	*m*(ZnO):*m*(BC)	*n*(ZnO):*n*(TEA)
a	0.25–0.50	1:5.5	1:1
b	0.50–0.80	1:5.5	1:1
c	0.50–0.80	1:11	1:1
d	0.50–0.80	1:16.5	1:1
e	0.50–0.80	1:11	2:1

^1^ The difference between sample a and sample b is the different bone char particle sizes. The difference between sample b, sample c, and sample d is the different mass ratios of ZnO to bone char. The difference between sample c and sample e is the different mole ratios of ZnO to triethylamine (TEA).

**Table 2:** The specific surface areas and average pore sizes of the ZnO/BC samples.

Sample	Specific Surface Area/m^2^ g^−1^	Average Pore Sizes/nm
a	105.9	12.15
b	106.7	12.10
c	100.1	13.64
d	103.4	12.12
e	108.2	13.61
